# Sex differences in skeletal muscle alterations in a model of colorectal cancer

**DOI:** 10.14814/phy2.14391

**Published:** 2020-03-13

**Authors:** Angela C. Greenman, Dawn M. Albrecht, Richard B. Halberg, Gary M. Diffee

**Affiliations:** ^1^ Balke Biodynamics Laboratory Department of Kinesiology University of Wisconsin Madison WI USA; ^2^ McArdle Laboratory for Cancer Research Department of Oncology School of Medicine and Public Health University of Wisconsin Madison WI USA; ^3^ Division of Gastroenterology and Hepatology Department of Medicine School of Medicine and Public Health University of Wisconsin Madison WI USA; ^4^ Carbone Cancer Center University of Wisconsin Madison WI USA

**Keywords:** Cancer cachexia, fatigability, muscle function, sex differences

## Abstract

Cancer cachexia is the loss of lean muscle mass with or without loss of fat mass that is often highlighted by a progressive loss of skeletal muscle mass and function. The mechanisms behind the cachexia‐related loss of skeletal muscle are poorly understood, including cachexia‐related muscle functional impairments. Existing models have revealed some potential mechanisms, but appear limited to how the cancer develops and the type of tumors that form. We studied the C57BL6/J (B6) *Apc^Min/+^* Tg::Fabp1‐Cre TG::PIK3ca* (CANCER) mouse. In this model, mice develop highly aggressive intestinal cancers. We tested whether CANCER mice develop cancer cachexia, if muscle function is altered and if sex differences are present. Both female and male mice, B6 (CONTROL) and CANCER mice, were analyzed to determine body weight, hindlimb muscle mass, protein concentration, specific force, and fatigability*.* Female CANCER mice had reduced body weight and hindlimb muscle mass compared with female CONTROL mice, but lacked changes in protein concentration and specific force. Male CANCER mice had reduced protein concentration and reduced specific force, but lacked altered body weight and muscle mass. There were no changes in fatigability in either group. Our study demonstrates that CANCER mice present an early stage of cachexia, have reduced specific force in male CANCER mice and develop a sex‐dependent cachexia phenotype. However, CANCER mice lack certain aspects of the syndrome seen in the human scenario and, therefore, using the CANCER mice as a preclinical model does not seem sufficient in order to maximize the translation of preclinical findings to humans.

## Graphical abstract

The mechanisms behind the progressive loss of skeletal muscle mass and function seen in patients with cancer cachexia are still largely unknown, thus we tested whether a new murine model of colorectal cancer could better represent the human condition. We concluded that although the tumor‐bearing model tested had reductions in body weight, muscle mass, specific force and a sex‐specific phenotype, the model tested had a limited lifespan and ultimately impaired the ability to translate findings to the human condition.

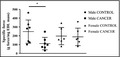

## 1 INTRODUCTION

Cachexia is a progressive syndrome involving the loss of skeletal muscle mass, with or without the loss of adipose tissue, due to an underlying disease, such as cancer. The severity of muscle mass loss, inflammation, loss of function and fatigue occurring in cancer cachexia is described as progressing through three stages of cancer cachexia: precachexia, cachexia, and refractory cachexia (Fearon et al., 2011). The mechanisms underlying the development and progression of cancer cachexia are yet to be fully understood, but cancer‐related systemic inflammation appears to negatively affect muscle mass (Argilés, Busquets, Toledo, & López‐soriano, 2009; Tisdale, 2009; VanderVeen, Fix, & Carson, 2017a). Approximately 60%–80% of cancer patients with advanced tumors suffer from cachexia (Tan & Fearon, 2008) and the syndrome is the cause of death in more than 20% of cancer patients (Tisdale, 2009). As the number of cancer survivors continues to rise (Howlader et al., 2016; Siegel, Miller, & Jemal, 2017), there is an increased urgency to better understand cancer cachexia and its long‐term impact on overall health and well‐being.

Previous studies of cancer cachexia, in both clinical and preclinical settings, have focused primarily on the ongoing loss of muscle mass with little attention paid to changes in muscle functional properties despite the fact that reduced quality of life and increased risk of mortality, common outcomes of cancer cachexia, are due to increased muscle fatigue and functional impairments (Murphy & Lynch, 2009). From the few preclinical studies measuring muscle function, via in vitro and in situ measures in various mouse models, tumor‐bearing mice tend to show reductions of specific force within various hindlimb skeletal muscles and the diaphragm, while also resulting in increased fatigability of tumor‐bearing muscles (Aulino et al., 2010; Murphy, Chee, Trieu, Naim, & Lynch, 2012; Roberts, Frye, Ahn, Ferreira, & Judge, 2013; VanderVeen, Hardee, Fix, & Carson, 2017b). Further determination of altered muscle functional properties accompanying the loss of muscle mass in cancer cachexia may lead to a more complete understanding of the cancer cachexia mechanism and therefore lead to potential therapeutic targets.

The most commonly studied animal model of cachexia, the Colon‐26 (C26) mouse (Aulino et al., 2010; Roberts et al., 2013), develops tumors following subcutaneous injection or grafting of C26 carcinoma cells into the flank of CD2F1or BALB/c mice (Aulino et al., 2010; Ballarò, Costelli, & Penna, 2016; Talbert, Metzger, He, & Guttridge, 2014). C26 mice experience rapid and drastic losses of skeletal muscle mass resulting from the extraneous inoculations or grafts and, therefore, may not appropriately model how cancer develops nor the progressive nature of cachexia in humans (Fearon et al., 2011; Talbert et al., 2019). An autochthonous model would likely more fully mimic the human condition. The *Apc^Min/+^* mouse spontaneously develops intestinal polyps, and has also been used to study cancer cachexia (Hetzler, Hardee, LaVoie, Murphy, & Carson, 2017; Mehl, Davis, Berger, & Carson, 2005). *Apc^Min/+^*mice develop cachexia over a greater time period (between 14 and 24 weeks of age (Mehl et al., 2005)) compared with the C26 mouse (Moser, Pitot, & Dove, 1990). In addition, *Apc^Min/+^*mice are often categorized by the amount of body weight lost (White et al., 2011), better representing the progressive nature of the syndrome (Fearon et al., 2011). Despite these advantages, there are some disadvantages of the *Apc^Min/+^*mouse as a model for human tumor development. For example, *Apc^Min/+^*mice typically develop several benign adenomas within the small intestine (Su et al., 1992), which may not adequately represent cancer cachexia in humans, who develop cachexia primarily as a result of solid tumors, such as carcinomas (e.g., adenocarcinomas) (Tan & Fearon, 2008).

The tumor‐bearing *Apc^Min/+^* Tg::Fabp1‐Cre TG::PIK3ca* (CANCER) mouse has been used to study colon cancer development, progression, and response to therapy (Deming et al., 2014; Foley et al., 2017). In these mice, tumors most often develop following the spontaneous loss of the *Apc* wild‐type allele. Some of these tumors also express an activated form of the PI3K kinase because Cre recombinase is expressed mosaically and removes a STOP sequence upstream of PIK3ca* transgene (Deming et al., 2014). These genetic alternations are relevant as both *APC* and *PIK3CA* mutations are commonly present in human colorectal cancers (Samuels et al., 2004). CANCER mice develop adenocarcinomas located within the small intestine and colon that are highly aggressive and sometimes metastasize to regional lymph nodes and other organs (Deming et al., 2014). We sought to determine whether CANCER mice could better represent how cancer cachexia develops in colorectal cancer patients.

In addition, it has been suggested that there are sex differences in both how cachexia develops and in the severity of the loss of muscle mass and function (Montalvo, Counts, & Carson, 2018), but few studies have directly addressed these sex differences. Female patients and rodents with cancer appear to benefit from the anti‐inflammatory effects of estrogen (Hetzler et al., 2015, 2017; Koo et al., 2008), which corresponds with data indicating that male cancer patients lost more body weight, muscle mass and have higher rates of mortality compared with their female counterparts (Baracos, Reiman, Mourtzakis, Gioulbasanis, & Antoun, 2010; Hendifar et al., 2009).

In this study, we aimed to determine whether (a) CANCER mice develop a cancer cachexia phenotype seen in humans, (b) muscle function is altered in these tumor‐bearing mice, and (c) sex differences in the degree of cachexia exist when comparing female versus male CANCER mice. We tested these aims by comparing body weight, muscle mass, protein concentration, specific force, and fatigue between female C57BL6/J (CONTROL) and female CANCER mice and also between male CONTROL and male CANCER mice. We hypothesized that 1) CANCER mice would develop cachexia and model the human condition, 2) CANCER mice, regardless of sex, would have reduced specific force and increased fatigability, and 3) male CANCER mice would exhibit a more severe cancer cachexia phenotype.

## 2 METHODS

### 2.1 Experimental animals

B6 female (*n* = 11) and male (*n* = 15) CANCER mice and B6 female (*n* = 9) and male (*n* = 9) age‐matched CONTROL mice were housed under protocols approved by the Institutional Animal Care and Use Committee at the University of Wisconsin (Madison, WI). Both female and male mice were studied to determine whether sex differences exist in the CANCER mouse model. CANCER mice, *Apc^Min/+^*; Fabp1‐Cre 1Jig; Gt(ROSA)26Sor^tm7(Pik3ca*,EGFP)Rsky^, were generated as previously described (Deming et al., 2014). CANCER mice spontaneously developed several tumors located within the colon due to the presence of tissue‐specific Cre recombinase expressed mosaically from the Fabp1‐Cre transgene in the distal sections of the small intestine and colon. Cre recombinase induced the deletion of a stop sequence, allowing Pik3ca* to be expressed. All mice were housed in climate‐controlled rooms with 12‐hr light/dark cycles and given standard rodent chow and water ad libitum. After weaning, the body weight of CANCER mice was measured weekly until euthanasia. Tumor mice were killed when moribund, which occurred between 50 and 70 days of age owing to advanced disease. Mice were considered moribund when they exhibited signs of anemia, which is secondary to bleeding intestinal tumors, or from obstructive enteropathy due to large colon tumors.

### 2.2 Hindlimb Dissection & Muscle Function Protocol

Hindlimb skeletal muscles (extensor digitorum longus (EDL), gastrocnemius (GAS), plantaris (PL), soleus (SOL), and tibialis anterior (TA)) were quickly excised and weighed upon euthanasia. Muscles not used for contractile measurements were quick frozen in liquid nitrogen and stored at −80°C for subsequent analysis. The tibia was dissected to determine tibia length, an indicator of animal body size that is independent of changes in muscle and fat mass.

**Figure 1:**
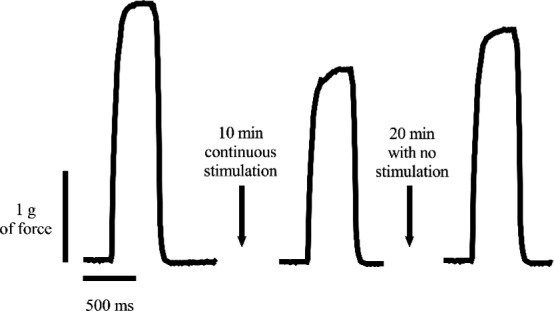
Representation of fatigability measured by continuously stimulating the EDL muscle for 10 min. Recovery of the EDL was defined as any increase in force following 20 min with no stimulation

After dissecting the hindlimb muscles, the EDL was attached to a contractile apparatus capable of measuring force (Aurora Scientific Model 801C) via hooks tied to the proximal and distal tendons. Muscles were perfused at room temperature with oxygenated (95% O_2_, 5% CO_2_) Tyrode's solution (NaCl 145 mM, KCl 5 mM, CaCl_2_ 2mM, MgCl_2_ 0.5 mM, NaH_2_PO_4_ 0.4 mM, NaHCO_3_ 24 mM, EDTA 0.1 mM, Glucose 10 mM) throughout the experiment. Muscles were electrically stimulated by two parallel platinum electrodes. Maximal twitch and tetanic force optimal length was determined in each muscle by adjusting the length of the muscle until twitch force reached its maximum, as described previously (Tetri et al., 2018). After determining maximal force, there was a 15‐min period of rest until fatigability was tested. The EDL was stimulated at 100 Hz for 500 ms every 5 s. The force from eight tetanic stimulations were averaged to assess maximal tetanic force production. The EDL underwent continuous stimulation for 10 min, and then the maximal tetanic force was measured again. Force after 10 min of continuous stimulation was calculated as a percent of maximum force, and this was termed the “fatigue force.” The EDL was allowed to recover for 20 min with no stimulation and force was once again measured to ensure that the muscle remained viable. Although force and fatigability tests vary greatly throughout the literature, we modeled our protocol to correspond with other cancer cachexia functional protocols (Murphy et al., 2012; Roberts et al., 2013). Fatigability was defined as the decline in tetanic force following 10 min of continuous stimulation. A representative test is shown in Figure 1. Following fatigue measurements, fatigued EDL muscles were weighed, quick frozen in liquid nitrogen and stored at −80°C.

### 2.3 Protein quantification

Previously quick frozen in liquid nitrogen and stored at −80°C hindlimb muscles (EDL, GAS, and TA) were homogenized in nonidet P‐40 lysis buffer. Total protein concentration (mg protein/mg muscle mass) was determined using the Bradford method (Bradford, 1976).

### 2.4 Statistical analysis

A Shapiro–Wilk test was completed to test for normality between groups. Based upon a non‐normal distribution of hindlimb muscle mass, a two‐tailed Wilcoxon rank‐sum test was used to compare muscle mass differences between CONTROL and CANCER mice: female CONTROL mice versus female CANCER mice and male CONTROL mice versus male CANCER mice. Student's *t*‐tests were used for all other group comparisons. Correlations were determined by Pearson's correlation and linear regression was used to determine significant relationships between the outcome variables. All statistical analyses were performed using a statistical program called SPSS. Significance was set at *p* ≤ .05 and all values presented as means ± *SD*.

## 3 RESULTS

### 3.1 Animal characteristics

**Figure 2:**
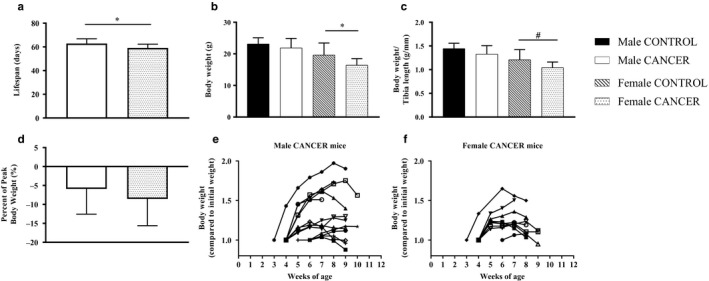
Lifespan and body weight analysis. (a) Lifespan comparison between male and female CANCER mice. (b) Body weight and (c) body weight to tibia length ratio compared between sex and genotype. (d) Final body weight compared with peak body weight in male CANCER and female CANCER mice. Body weight compared to initial body weight throughout the lifespan of each (e) male and (f) female CANCER mouse. Sample sizes for male CONTROL, male CANCER, female CONTROL, and female CANCER mice body weight measurements were 9, 13–15, 9, and 9–11, respectively. * represents *p* ≤ .05 and # represents *p* = .08

To determine whether CANCER mice develop cancer cachexia, we measured body weight and hindlimb muscle mass in both CANCER and CONTROL mice. Both female and male CANCER mice had large tumors present (1–5 visible) within the intestinal epithelium upon dissection. Female CANCER mice had a significantly shorter lifespan compared with male CANCER mice (Figure 2a). Additionally, Female CANCER mice had significantly reduced body weight compared with female CONTROL mice (Figure 2b). When body weight was normalized to tibia length, female CANCER mice still trended smaller compared with female CONTROL mice, but was not statistically significant (*p* = .08, Figure 2c). Male CANCER mice did not differ from male CONTROL mice when analyzing body weight and body weight to tibia length (Figure 2b‐c). When body weight was analyzed as a ratio of the final body weight compared with the highest body weight achieved during 8 weeks of measurement, both male and female CANCER groups had reductions in body weight (Figure 2d).

### 3.2 Skeletal muscle mass

**Figure 3:**
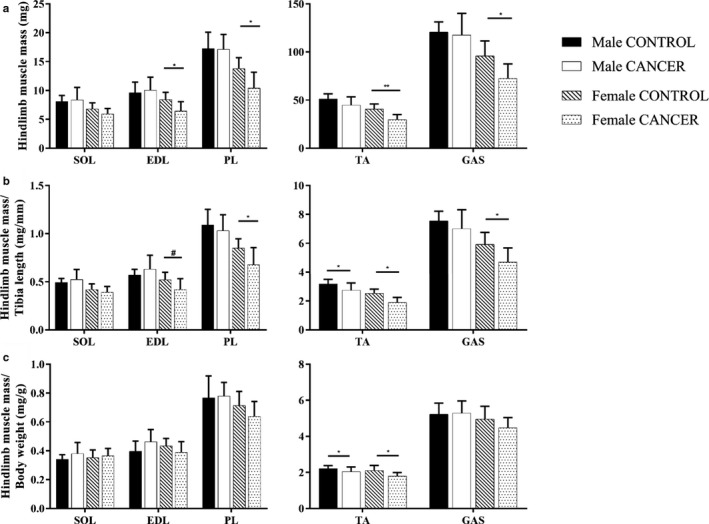
Hindlimb skeletal muscle mass. (a) Absolute hindlimb muscle mass, (b) muscle mass to tibia length ratio and (c) muscle mass to body weight ratio compared between sex and genotype. Sample sizes for male CONTROL, male CANCER, female CONTROL, and female CANCER mice per hindlimb muscle were 8–9, 12–14, 9, and 9–11, respectively. * represents *p* ≤ .05, ** represents *p* ≤ .005 and # represents *p* < .07

Female CANCER mice had a reduction in absolute mass of several hindlimb muscles (EDL, GAS, PL and TA) compared to female CONTROL mice (Figure 3a). Female CANCER mice had reduced muscle mass to tibia length ratio in the EDL (*p* = .06), TA, PL, and GAS, but not within the SOL (Figure 3b). There were no differences in absolute hindlimb muscles mass between male CANCER and male CONTROL mice (Figure 3a). Male CANCER mice had a significant reduction in TA mass to tibia length compared with male CONTROL mice (*p* = .03), but the other hindlimb muscles failed to show any differences (Figure 3b). Lastly, both female and male CANCER mice had reduced TA mass to body weight ratios (Figure 3c), but other hindlimb muscle mass to body weight ratios did not differ between CANCER and CONTROL mice, regardless of sex.

### 3.3 Protein concentration

Myofibrillar proteins contribute to a significant proportion of skeletal muscle mass. We measured the protein concentration (mg protein/mg muscle mass) of the EDL, GAS, and TA to determine if either proteolysis or declines in protein synthesis were occurring prior to or during declines in skeletal muscle mass. We were interested in measuring the protein concentration of the EDL to determine whether protein quantity was positively associated with EDL‐specific force. In addition to measuring EDL protein concentration, we measured TA protein concentration because both female and male CANCER mice showed evidence of TA atrophy, normalized to tibia length and body weight. The generous size of the GAS made it highly feasible to study GAS protein concentration.

**Figure 4:**
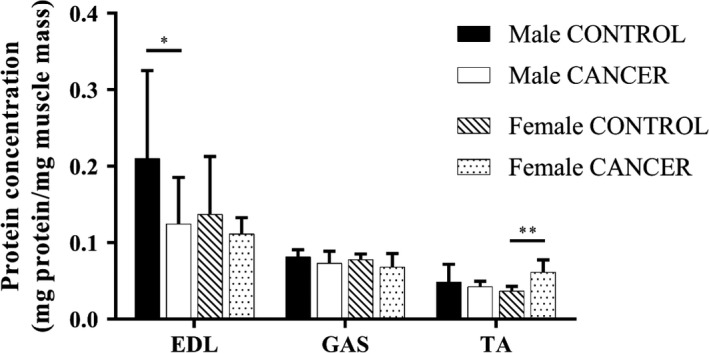
Protein Concentration of hindlimb skeletal muscles (EDL, GAS, and TA) compared between sex and genotype. Sample sizes for male CONTROL, male CANCER, female CONTROL, and female CANCER mice per hindlimb muscle were 8, 11–13, 7, and 6–8, respectively. * represents *p* ≤ .05 and ** represents *p* ≤ .005

There was no significant difference in the EDL protein concentration measured in female CONTROL mice and female CANCER mice, but male CANCER mice had reduced EDL protein concentration compared with male CONTROL mice (*p* < .05; Figure 4). In male CANCER mice, there were reductions in EDL protein concentration despite the maintenance of EDL muscle mass, shown previously in Figure 3a–c. Female CANCER mice had increased TA protein concentration compared with female CONTROL mice (*p* < .005; Figure 4), despite the reduction in TA mass in the female CANCER group shown in Figure 3a–c. There was no significant difference in the TA protein concentration between male CANCER and CONTROL mice. Lastly, GAS protein concentration was not statistically different in either female or male groups.

### 3.4 Specific force

**Figure 5:**
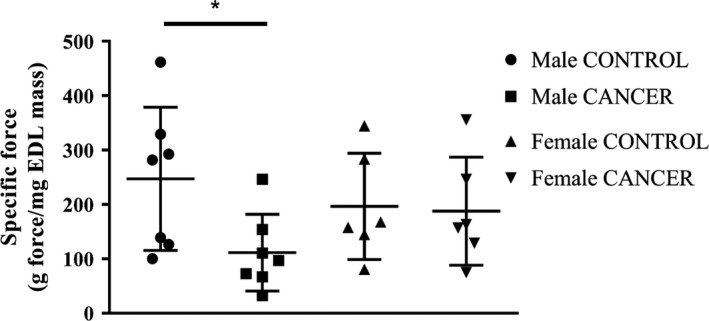
Specific force of the EDL hindlimb muscle. *n* = 7 male mice and *n* = 6 female mice per group. * represents *p* < .05

Functional measurements, specific force and fatigue, were measured in the EDL muscle to determine whether there were changes in function were occurring at the cellular level beyond changes in muscle mass or protein. The EDL was utilized for in vitro functional measurements due to its small size and fiber type composition. Glycolytic muscles, such as the EDL, might be sensitive to changes associated with cancer cachexia (Carson, Hardee, & VanderVeen, 2016). There was no difference in specific force between female CANCER mice and female CONTROL mice (Figure 5). Male CANCER mice produced significantly less specific force (EDL force normalized to EDL mass) compared with male CONTROL mice (*p* < .03; Figure 5).

### 3.5 Fatigability

**Figure 6:**
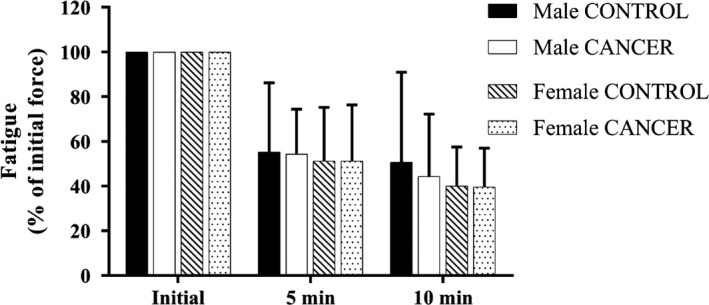
Fatigability of the EDL. There were no significant differences between groups at either 5 or 10 min of fatiguing stimulation. *n* = 7 male mice and *n* = 6 female mice per group

There were no differences in fatigability, the amount of force generated relative to the initial force, between CANCER and CONTROL mice at 5 min and 10 min of continuously stimulation (Figure 6), regardless of sex. All EDL muscles recovered from 10 min of fatiguing stimulation.

## 4 DISCUSSION

To our knowledge, this is the first study to quantify skeletal muscle atrophy and muscle function in the CANCER mouse. The previously used *Apc^Min/+^*mouse results in a high prevalence of adenomas and thus may not accurately represent the cancer cachexia phenotype in humans. CANCER mice carry genetic alterations in genes that are commonly mutated in human colorectal cancers (Deming et al., 2014). Patients with solid tumors (e.g., carcinomas) have been shown to experience the greatest amount of body weight loss (Tan & Fearon, 2008). We utilized CANCER mice to study cancer cachexia to employ a tumor model that more closely represents the human scenario. We concluded that this model of colon cancer presented an early stage of cancer cachexia with body weight loss relative to peak body weight, muscle‐specific loss in mass‐ and a sex‐dependent reduction in force production. Despite this, we concluded that this mouse model might not be the ideal preclinical model for closely mimicking the human condition based primarily upon the short lifespan of CANCER mice owing to the highly aggressive cancers.

It has been suggested that cancer cachexia affects females and males differently both in animal models and human subjects (Clocchiatti, Cora, Zhang, & Dotto, 2016; Norman et al., 2012). Hetzler and colleagues found that male *Apc^Min/+^*cachectic mice have greater reductions in body weight compared with female *Apc^Min/+^*cachectic mice, despite a similar tumor burden (Hetzler et al., 2015). In this study, female CANCER mice exhibited more severe reductions in body weight and muscle mass compared with male CANCER mice, whereas male CANCER mice had a greater reduction in EDL protein concentration and specific force. Our results might differ from previous studies owing to different animal models being studied, further indicating the importance of modeling how cancer spontaneously develops and progresses in the human condition.

As in healthy individuals, skeletal muscle mass is influenced by sex hormones. Androgens have a large anabolic effect in males in part due to augmented expression of insulin‐like growth factor‐1 in skeletal muscle which activates the protein synthesis pathway (Anderson, Liu, & Garcia, 2017). Men with cancer have a higher prevalence of hypogonadism compared with the general population and it has been suggested that cancer patients with hypogonadism have both reduced quality of life and survival (Burney & Garcia, 2012). In addition, estrogen appears to have a protective effect, increasing the survival of young women with colorectal cancer (Koo et al., 2008). Estrogen has been found to have both an anti‐inflammatory effect and to reduce protein degradation via inhibition of the proteasome pathway (Anderson et al., 2017). Hetzler and colleagues found that cachectic female *Apc^Min/+^*mice had reduced uterine weight, which corresponded with a reduction in estrogen levels and a more severe cachexia phenotype (Hetzler et al., 2017). It is plausible that the observed sex differences seen in the CANCER model could be due, at least in part, to cancer‐related hormonal changes. Although some investigators have begun studying these differences, sexual dimorphism in cancer cachexia is still largely unexplored, especially when considering contractile properties.

To date, the cachexia literature has primarily focused on the effects of cancer on skeletal muscle mass. Our study aimed to address a gap in the literature by also measuring muscle function in tumor‐bearing mice. We found a reduction in specific force (force normalized to EDL mass) in male CANCER mice compared with male CONTROL mice (*p* < .03). These findings are consistent with other studies showing that hindlimb‐specific force is reduced in male tumor‐bearing mice (Roberts et al., 2013; VanderVeen, Hardee, et al., 2017). To our knowledge, this is the first study showing no change in specific force in tumor‐bearing female mice. In addition to the decline in EDL‐specific force measured in male CANCER mice, there was also a reduction in the EDL protein concentration in the male CANCER mice compared with male CONTROL mice, although EDL force and protein concentration were not correlated together. The reduction in protein concentration matched with a decrease in specific force might indicate that contractile proteins, such as myosin (Diffee, Kalfas, Al‐Majid, & McCarthy, 2002), are affected by cancer cachexia.

We initially hypothesized that all CANCER mice would have increased fatigability compared with CONTROL mice due to previous evidence that cancer cachexia is associated with reductions in skeletal muscle mitochondrial function (Carson et al., 2016). Instead, we found that there was no difference in fatigability between CANCER mice and CONTROL mice, regardless of sex. Whereas other studies have suggested that muscles from C26 and *Apc^Min/+^*mice have increased fatigability (Roberts et al., 2013; VanderVeen, Hardee, et al., 2017). The fatigue protocol used in this study has previously been used to study fatigability and mitochondrial function in premature rats exposed to postnatal hyperoxia and it was found that increased fatigability, using this protocol, was correlated with mitochondrial dysfunction (Tetri et al., 2018). Thus, the lack of cachexia‐related changes in fatigability measured in our study suggests a lack of significant changes in mitochondrial function in these muscles. Our results may differ from previous works due to different time points and models of cancer cachexia being studied. For example 18‐week‐old *Apc^Min/+^*mice had increased fatigability of the TA, but 12‐week‐old *Apc^Min/+^*mice did not show any differences in fatigability when compared with nontumor‐bearing, age‐matched littermates (VanderVeen, Hardee, et al., 2017). In this study, we utilized approximately 8‐week‐old mice to measure fatigue, thus it is possible there was not enough time for mitochondrial changes to accumulate and affect fatigability in CANCER mice. In addition, previous muscle functional studies have used C26 and *Apc^Min/+^*mice, which, as previously mentioned, develop cancer differently (e.g., inoculation versus. genetic mutations) and, therefore, might alter fatigue differently.

Fearon and colleagues defined cancer cachexia as a progressive syndrome categorized into three stages: precachexia, cachexia, and refractory cachexia (Fearon et al., 2011). Criteria for categorization includes food intake, catabolic drive, muscle mass, strength, psychosocial effects, and functional measures. In this study, there appears to be different amounts of the muscle mass loss and functional impairments when compared with other mouse models of cancer cachexia (e.g., C26 and *Apc^Min/+^*mice), specifically when considering the lack of body weight and muscle mass loss observed in male CANCER mice. These different results might be due to various stages of the progressive syndrome being studied. We suggest that the CANCER mouse best represented the precachexia stage. In addition to studying various stages, current animal models used to study cancer cachexia develop cancer differently (e.g., inoculations versus mutated genes). Studying various stages and types of cancer development might affect the cachexia phenotype being studied. Thus, future works should better identify if the varying stages and preclinical models studied affect our understanding of the syndrome in humans and the ability to translate findings to humans.

We originally hypothesized that declines in skeletal muscle mass would also lead to declines in the protein concentration of those muscles. However, female CANCER mice had reduced muscle mass without congruent declines in protein concentration of the EDL and GAS. Female CANCER mice even had augmented protein concentration within the TA muscle compared with female CONTROL mice. In addition, male CANCER mice had reduced protein concentration of the EDL compared with male CONTROL mice, but the declines in protein concentration measured were independent of any significant declines in skeletal muscle mass of the EDL. Muscle mass is determined by more than just the mass of myofibril proteins, making it plausible that there were other variables, such as fat or connective tissue, contributing to muscle mass and protein concentration measurements in this study.

Although there are advantages to utilizing *Apc^Min/+^* Tg::Fabp1‐Cre TG::PIK3ca* (CANCER) mice to study cachexia, this animal model does have limitations. CANCER mice quickly develop aggressive cancer and succumb to the disease often by 60 days of age, which did not allow for studying the progressive nature of cancer cachexia. This model mimics human colorectal cancer better than others that have been employed because the tumors progress to a malignant state owing to alterations in two driver genes. However, the rapid rate of progression might limit the time necessary for cachectic changes as manifested in muscle function or body weight to occur. In addition, while CANCER mice carry mutations in the *Apc* and *Pik3ca* genes, and the homologs of these genes are often mutated in human colorectal cancers, many other genes have also been associated with the disease. There is a need for other animal models that might better mimic the timing and other characteristics of human cancer development.

## 5 CONCLUSION

In conclusion, our study showed that the CANCER mice may present an early stage of cancer cachexia, had altered muscle function and developed sex differences. But, despite these outcomes, findings from CANCER mice are still limited in how well this preclinical model translates to the human condition. Female CANCER mice experienced reductions in body weight and muscle mass compared with female CONTROL mice, whereas male CANCER mice had reduced protein concentration and skeletal muscle function compared with male CONTROL mice. Fatigability did not change in either group. We suggest that sex differences observed in cancer cachexia may be dependent on, not only hormonal changes seen in the literature, but also the various animal models being studied (the type of mutations occurring, the location of the tumors and the age of the animals being studied). Despite efforts to model the human condition with CANCER mice, the limited lifespan on the CANCER mice seems to limit the ability to translate our findings to humans. Future works should continue to focus on the variable results found between animal models, when measuring outcome variables such as skeletal muscle mass and function, and how well these data translate to patients within various different stages of cancer cachexia.

## CONFLICT OF INTEREST

The authors have no conflicts of interest to disclose.

## References

[phy214391-bib-0001] Anderson, L. J. , Liu, H. , & Garcia, J. M. (2017). Sex Differences in Muscle Wasting, in: Sex and Gender Factors Affecting Metabolic Homeostasis, Diabetes and Obesity, Advances in Experimental Medicine and Biology. Cham: Springer, pp. 153–197. 10.1007/978-3-319-70178-3_9 29224095

[phy214391-bib-0002] Argilés, J. M. , Busquets, S. , Toledo, M. , & López‐soriano, F. J. (2009). The role of cytokines in cancer cachexia. Current Opinion in Supportive and Palliative Care, 3, 263–268. 10.1097/SPC.0b013e3283311d09 19713854

[phy214391-bib-0003] Aulino, P. , Berardi, E. , Cardillo, V. M. , Rizzuto, E. , Perniconi, B. , Ramina, C. , … Coletti, D. (2010). Molecular, cellular and physiological characterization of the cancer cachexia‐inducing C26 colon carcinoma in mouse. BMC Cancer, 10, 363 10.1186/1471-2407-10-363 20615237 PMC2912868

[phy214391-bib-0004] Ballarò, R. , Costelli, P. , & Penna, F. (2016). Animal models for cancer cachexia. Current Opinion in Supportive and Palliative Care, 10, 281–287. 10.1097/SPC.0000000000000233 27454355

[phy214391-bib-0005] Baracos, V. E. , Reiman, T. , Mourtzakis, M. , Gioulbasanis, I. , & Antoun, S. (2010). Body composition in patients with non‐small cell lung cancer: A contemporary view of cancer cachexia with the use of computed tomography image analysis. American Journal of Clinical Nutrition, 91, 1133S–1137S. 10.3945/ajcn.2010.28608C 20164322

[phy214391-bib-0006] Bradford, M. M. (1976). A rapid and sensitive method for the quantitation of microgram quantities of protein utilizing the principle of protein‐dye binding. Analytical Biochemistry, 72, 248–254. 10.1016/0003-2697(76)90527-3 942051

[phy214391-bib-0007] Burney, B. O. , & Garcia, J. M. (2012). Hypogonadism in male cancer patients. Journal of Cachexia, Sarcopenia and Muscle, 3, 149–155. 10.1007/s13539-012-0065-7 PMC3424192 22528986

[phy214391-bib-0008] Carson, J. A. , Hardee, J. P. , & VanderVeen, B. N. (2016). The emerging role of skeletal muscle oxidative metabolism as a biological target and cellular regulator of cancer‐induced muscle wasting. Seminars in Cell & Developmental Biology, 54, 53–67. 10.1016/j.semcdb.2015.11.005 26593326 PMC4867246

[phy214391-bib-0009] Clocchiatti, A. , Cora, E. , Zhang, Y. , & Dotto, G. P. (2016). Sexual dimorphism in cancer. Nature Reviews Cancer, 16, 330–339. 10.1038/nrc.2016.30 27079803

[phy214391-bib-0010] Deming, D. A. , Leystra, A. A. , Nettekoven, L. , Sievers, C. , Miller, D. , Middlebrooks, M. , … Halberg, R. B. (2014). PIK3CA and APC Mutations are Synergistic in the Development of Intestinal Cancers. Oncogene, 33, 2245–2254. 10.1038/onc.2013.167 23708654 PMC3883937

[phy214391-bib-0011] Diffee, G. M. , Kalfas, K. , Al‐Majid, S. , & McCarthy, D. O. (2002). Altered expression of skeletal muscle myosin isoforms in cancer cachexia. American Journal of Physiology. Cell Physiology, 283, C1376–1382. 10.1152/ajpcell.00154.2002 12372798

[phy214391-bib-0012] Fearon, K. , Strasser, F. , Anker, S. D. , Bosaeus, I. , Bruera, E. , Fainsinger, R. L. , … Baracos, V. E. (2011). Definition and classification of cancer cachexia: An international consensus. The Lancet Oncology, 12, 489–495. 10.1016/S1470-2045(10)70218-7 21296615

[phy214391-bib-0013] Foley, T. M. , Payne, S. N. , Pasch, C. A. , Yueh, A. E. , Hey, D. R. V. D. , Korkos, D. P. , … Deming, D. A. (2017). Dual PI3K/mTOR Inhibition in Colorectal Cancers with APC and PIK3CA Mutations. Molecular Cancer Research, 15, 317–327. 10.1158/1541-7786.MCR-16-0256 28184015 PMC5550373

[phy214391-bib-0014] Hendifar, A. , Yang, D. , Lenz, F. , Lurje, G. , Pohl, A. , Lenz, C. , … Lenz, H.‐J. (2009). Gender Disparities in Metastatic Colorectal Cancer Survival. Clinical Cancer Research, 15, 6391–6397. 10.1158/1078-0432.CCR-09-0877 19789331 PMC2779768

[phy214391-bib-0015] Hetzler, K. L. , Hardee, J. P. , LaVoie, H. A. , Murphy, E. A. , & Carson, J. A. (2017). Ovarian function’s role during cancer cachexia progression in the female mouse. American Journal of Physiology‐Endocrinology and Metabolism, 312, E447–E459. 10.1152/ajpendo.00294.2016 28292759 PMC5451525

[phy214391-bib-0016] Hetzler, K. L. , Hardee, J. P. , Puppa, M. J. , Narsale, A. A. , Sato, S. , Davis, J. M. , & Carson, J. A. (2015). Sex Differences in the Relationship of IL‐6 Signaling to Cancer Cachexia Progression. Biochimica Et Biophysica Acta, 1852, 816–825. 10.1016/j.bbadis.2014.12.015 25555992 PMC4372501

[phy214391-bib-0017] Howlader, N. , Noone, A. , Krapcho, M. , Miller, D. , Bishop, K. , Kosary, C. , …Cronin, K. 2016 SEER Cancer Statistics Review, 1975–2014, National Cancer Institute.

[phy214391-bib-0018] Koo, J. H. , Jalaludin, B. , Wong, S. K. C. , Kneebone, A. , Connor, S. J. , & Leong, R. W. L. (2008). Improved survival in young women with colorectal cancer. American Journal of Gastroenterology, 103, 1488–1495. 10.1111/j.1572-0241.2007.01779.x 18510616

[phy214391-bib-0019] Mehl, K. A. , Davis, J. M. , Berger, F. G. , & Carson, J. A. (2005). Myofiber degeneration/regeneration is induced in the cachectic ApcMin/+ mouse. Journal of Applied Physiology, 99, 2379–2387. 10.1152/japplphysiol.00778.2005 16288100

[phy214391-bib-0020] Montalvo, R. N. , Counts, B. R. , & Carson, J. A. (2018). Understanding sex differences in the regulation of cancer‐induced muscle wasting. Current Opinion in Supportive and Palliative Care, 12, 394–403. 10.1097/SPC.0000000000000380 30102621 PMC6239206

[phy214391-bib-0021] Moser, A. R. , Pitot, H. C. , & Dove, W. F. (1990). A dominant mutation that predisposes to multiple intestinal neoplasia in the mouse. Science, 247, 322–324. 2296722 10.1126/science.2296722

[phy214391-bib-0022] Murphy, K. T. , Chee, A. , Trieu, J. , Naim, T. , & Lynch, G. S. (2012). Importance of functional and metabolic impairments in the characterization of the C‐26 murine model of cancer cachexia. Disease Models & Mechanisms, 5, 533–545. 10.1242/dmm.008839 22563056 PMC3380716

[phy214391-bib-0023] Murphy, K. T. , & Lynch, G. S. (2009). Update on emerging drugs for cancer cachexia. Expert Opinion on Emerging Drugs, 14, 619–632. 10.1517/14728210903369351 19860537

[phy214391-bib-0024] Norman, K. , Stobäus, N. , Reiß, J. , Schulzke, J. , Valentini, L. , & Pirlich, M. (2012). Effect of sexual dimorphism on muscle strength in cachexia. Journal of Cachexia, Sarcopenia and Muscle, 3, 111–116. 10.1007/s13539-012-0060-z PMC3374022 22476918

[phy214391-bib-0025] Roberts, B. M. , Frye, G. S. , Ahn, B. , Ferreira, L. F. , & Judge, A. R. (2013). Cancer cachexia decreases specific force and accelerates fatigue in limb muscle. Biochemical and Biophysical Research Communications, 435, 488–492. 10.1016/j.bbrc.2013.05.018 23673294 PMC3708303

[phy214391-bib-0026] Samuels, Y. , Wang, Z. , Bardelli, A. , Silliman, N. , Ptak, J. , Szabo, S. , … Velculescu, V. E. (2004). High frequency of mutations of the PIK3CA gene in human cancers. Science, 304, 554 10.1126/science.1096502 15016963

[phy214391-bib-0027] Siegel, R. L. , Miller, K. D. , & Jemal, A. (2017). Cancer Statistics, 2017. CA: A Cancer Journal for Clinicians, 67, 7–30. 10.3322/caac.21387 28055103

[phy214391-bib-0028] Su, L. K. , Kinzler, K. W. , Vogelstein, B. , Preisinger, A. C. , Moser, A. R. , Luongo, C. , … Dove, W. F. (1992). Multiple intestinal neoplasia caused by a mutation in the murine homolog of the APC gene. Science, 256, 668–670. 1350108 10.1126/science.1350108

[phy214391-bib-0029] Talbert, E. E. , Cuitiño, M. C. , Ladner, K. J. , Rajasekerea, P. V. , Siebert, M. , Shakya, R. , … Guttridge, D. C. (2019). Modeling Human Cancer‐induced Cachexia. Cell Reports, 28, 1612–1622.e4. 10.1016/j.celrep.2019.07.016 31390573 PMC6733019

[phy214391-bib-0030] Talbert, E. E. , Metzger, G. A. , He, W. A. , & Guttridge, D. C. (2014). Modeling human cancer cachexia in colon 26 tumor‐bearing adult mice. Journal of Cachexia, Sarcopenia and Muscle, 5, 321–328. 10.1007/s13539-014-0141-2 PMC4248405 24668658

[phy214391-bib-0031] Tan, B. H. L. , & Fearon, K. C. H. (2008). Cachexia: Prevalence and impact in medicine. Current Opinion in Clinical Nutrition and Metabolic Care, 11, 400–407. 10.1097/MCO.0b013e328300ecc1 18541999

[phy214391-bib-0032] Tetri, L. H. , Diffee, G. M. , Barton, G. P. , Braun, R. K. , Yoder, H. E. , Haraldsdottir, K. , … Goss, K. N. (2018). Sex‐specific skeletal muscle fatigability and decreased mitochondrial oxidative capacity in adult rats exposed to postnatal hyperoxia. Frontiers in Physiology, 9, 10.3389/fphys.2018.00326 PMC5884929 29651255

[phy214391-bib-0033] Tisdale, M. J. (2009). Mechanisms of Cancer Cachexia. Physiological Reviews, 89, 381–410. 10.1152/physrev.00016.2008 19342610

[phy214391-bib-0034] VanderVeen, B. N. , Fix, D. K. , & Carson, J. A. (2017a). Disrupted Skeletal Muscle Mitochondrial Dynamics, Mitophagy, and Biogenesis during Cancer Cachexia: A Role for Inflammation [WWW Document]. Oxid. Med. Cell. Longev. https://www.hindawi.com/journals/omcl/2017/3292087/. 10.1155/2017/3292087 PMC5530417 28785374

[phy214391-bib-0035] VanderVeen, B. N. , Hardee, J. P. , Fix, D. K. , & Carson, J. A. (2017b). Skeletal muscle function during the progression of cancer cachexia in the male ApcMin/+ Mouse. Journal of Applied Physiology, jap.00897.2017, 10.1152/japplphysiol.00897.2017 PMC5899274 29122966

[phy214391-bib-0036] White, J. P. , Baynes, J. W. , Welle, S. L. , Kostek, M. C. , Matesic, L. E. , Sato, S. , & Carson, J. A. (2011). The regulation of skeletal muscle protein turnover during the progression of cancer cachexia in the ApcMin/+ Mouse. PLoS ONE, 6, e24650 10.1371/journal.pone.0024650 21949739 PMC3176277

